# Loss of NK Stimulatory Capacity by Plasmacytoid and Monocyte-Derived DC but Not Myeloid DC in HIV-1 Infected Patients

**DOI:** 10.1371/journal.pone.0017525

**Published:** 2011-03-08

**Authors:** Adel Benlahrech, Frances Gotch, Peter Kelleher, Steven Patterson

**Affiliations:** Department of Immunology, Imperial College London, Chelsea and Westminster Hospital, London, United Kingdom; Massachusetts General Hospital, United States of America

## Abstract

Dendritic cells (DC) are potent inducers of natural killer (NK) cells. There are two distinct populations in blood, myeloid (mDC) and plasmacytoid (pDC) but they can also be generated *In vitro* from monocytes (mdDC). Although it is established that blood DC are lost in HIV-1 infection, the full impact of HIV-1 infection on DC-NK cell interactions remains elusive. We thus investigated the ability of pDC, mDC, and mdDC from viremic and anti-retroviral therapy-treated aviremic HIV-1+ patients to stimulate various NK cell functions. Stimulated pDC and mdDC from HIV-1+ patients showed reduced secretion of IFN-α and IL-12p70 respectively and their capacity to stimulate expression of CD25 and CD69, and IFN-γ secretion in NK cells was also reduced. pDC activation of NK cell degranulation in response to a tumour cell line was severely reduced in HIV-1+ patients but the ability of mDC to activate NK cells was not affected by HIV-1 infection, with the exception of HLA-DR induction. No differences were observed between viremic and aviremic patients indicating that anti-retroviral therapy had minimal effect on restoration on pDC and mdDC-mediated activation of NK cells. Results from this study provide further insight into HIV-1 mediated suppression of innate immune functions.

## Introduction

Natural killer (NK) cells are the principal effectors of the innate system and play a pivotal role in tumour surveillance and anti-viral immunity [1], [2]. In the context of HIV-1 infection, NK cells may partially control the virus, especially at the early stages of HIV-1 infection before adaptive immunity is initiated [3]. NK cells have been shown to directly or indirectly kill HIV-1 infected cells as well as block HIV-1 entry and replication through secretion of a number of cytokines and chemokines including IFN-γ, TNF-α, and CCR5 binding CC chemokines [4]–[7].

Dendritic cells (DC) are antigen presenting cells which act as sentinels for the immune system by engulfing pathogens, processing and presenting them to CD4 and CD8 T cells. They express a variety of pattern recognition receptors including toll-like receptors (TLR) allowing recognition of a wide range of pathogens (reviewed in [8]). DC are a heterogeneous population of cells found in blood and most peripheral tissues, particularly at interfaces with the external environment. In humans, two major DC subtypes have been described in peripheral blood, myeloid (mDC) and plasmacytoid DC (pDC), serving different functions. mDC are thought to be the precursors of tissue DC and efficiently capture antigen for presentation and stimulation of CD4 and CD8 T cells. They express TLRs 1, 2, 3, 4, 5, 6, and 8 resulting in their ability to respond to stimulation with bacterial cell wall components and viral RNA [9], [10]. Whilst pDC can also present antigen to T cells, they uniquely secrete large amounts of IFN-α that contributes to anti-viral immunity [11], [12]. pDC express TLR-7 and TLR-9 and are therefore responsive to RNA viruses, and bacterial DNA containing unmethylated CpG sequences [10], [13].

DC are a rare cell type both in the periphery and in lymphoid organs, constituting about 1% of mononuclear leukocytes, which makes their study demanding. However, DC can be generated in large numbers *In vitro* by culturing peripheral monocytes in the presence of GM-CSF and IL-4 [14] and such cells have provided much of our current understanding of human DC biology.

Although, the principal function attributed to DC is their ability to prime, modulate, and maintain T and B cell responses, evidence collected over the last decade suggests that DC play an essential role in shaping NK cell-mediated immunity. Both *in vivo* [15], [16] and *in vitro* studies [17]–[20] have demonstrated that activated DC can stimulate activation, proliferation, IFN-γ production, and cytolytic activity of NK cells. The interactions between DC and NK cells are not unidirectional as shown by a number of reports [17], [18]. Activated NK cells can kill immature DC [19], [20] thereby providing a selection mechanism for DC that are competent at priming T cells. NK cells have also been shown to be capable of inducing maturation and type I polarisation of DC in the absence of TLR stimulation, which may be important in the initiation of adaptive immunity against transformed and tumourigenic cells (reviewed in [21]).

In the setting of HIV infection, several reports have identified both numerical and functional defects in the DC and NK cell compartments [22]–[29]. However little is known about the effect of HIV-1 infection on DC-NK bidirectional interplay. Two recent studies [30], [31] have addressed pDC-NK cell interactions during HIV-1 infection. Reitano et al found reduced amounts of IFN-α and TNF-α in CpG stimulated PBMC from untreated and HAART-treated patients, and impaired activation of NK cells, as indicated by CD69 expression, due to decreased levels of, and decreased responsiveness to, the pDC produced cytokines [31]. The study by Conry et al found that NK cells from untreated patients were defective in the pDC mediated IFN-γ production and killing activity, the former being due to both impaired pDC and NK function whilst the latter was mainly due to NK cell defects. Killing activity by NK cells was recovered in HAART-treated patients but IFN-γ production remained reduced [30]. Here we have further characterised the extent of pDC impairment by analysing several NK cell functions including expression of CD69 and CD25, IFN-γ production, and tumour killing potential in HAART- treated and untreated HIV-1 infected individuals. In addition, for the first time to our knowledge, we have analysed the function of mDC and mdDC from HIV-1 infected individuals in the activation of allogeneic NK cells.

## Materials and Methods

### Study subjects

The patient cohort consisted of 13 treatment-naïve and 12 HAART-treated HIV-1 infected individuals. Patients were selected randomly from those attending an HIV clinic at the Kobler centre, Chelsea and Westminster Hospital (London, United Kingdom). Untreated patients were not on anti-retroviral therapy and had CD4 T cell counts ranging from 41–1000 cells/µl (median of 255 cells/µl, Table 1) and plasma viral loads ranging from 202 to greater than 500,000 copies/ml (median of 14337 copies/ml, Table 1). All treated patients had been receiving HAART for at least 4.4 years. Their CD4 counts at the time of blood collection ranged from 185 to 1000 cells/µl (median of 500 cells/µl, Table 1) and they all had undetectable viral loads (<50 copies/ml). 12 control samples were obtained from HIV-1 seronegative individuals and single donor buffy coats (the National Blood Bank, Colindale, London). The latter were used to obtain large numbers of NK cells. Ethical approval and informed consent were obtained prior to blood donation.

**Table 1:** Clinical characteristics of HIV-1 patient cohorts.

Patient ID	Treatment history	Years on therapy	Age (years)	CD4 count (cell/µl)	Plasma VL(copies/ml)
Control group (n = 12)	Healthy controls	n.a.	Median (30) range (23–60)	n.t.	n.a.
N01	Naïve	n.a.	46	214	500000
N02	Naïve	n.a.	36	69	226208
N03	Naïve	n.a.	38	198	1163
N04	Naïve	n.a.	33	362	202
N05	Naïve	n.a.	41	350	7000
N06	Naïve	n.a.	42	460	324414
N07	Naïve	n.a.	40	1000	10000
N08	Naïve	n.a.	36	41	100389
N09	Naïve	n.a.	60	957	20497
N10	Naïve	n.a.	33	255	68342
N11	Naïve	n.a.	30	215	14337
N12	Naïve	n.a.	37	451	1480
N13	Naïve	n.a.	47	191	14069
**Median (range)**	**-**	**-**	**38 (30–60)**	**255 (41–1000)**	**14337 (202–500000)**
H01	HAART	5.5	41	500	<50
H02	HAART	4.4	54	390	<50
H03	HAART	5.4	50	1000	<50
H04	HAART	5.6	45	500	<50
H05	HAART	5.9	43	405	<50
H06	HAART	8.3	43	548	<50
H07	HAART	8.7	41	961	<50
H08	HAART	5.8	53	302	<50
H09	HAART	3.9	44	185	<50
H10	HAART	11.6	43	505	<50
H11	HAART	11.6	47	705	<50
H12	HAART	4.0	59	414	<50
**Median (range)**	**-**	**5.7 (3.9–11.6)**	**44.5 (41–59)**	**500 (185–1000)**	**<50**

n.a: not applicable, m: male, f: female, n.t. not tested.

### Cell isolations

60 ml of blood was collected from all participants in EDTA-coated Vacutainers. Peripheral blood mononuclear cells (PBMC) were isolated within 4 hours of blood collection by Ficoll-Histopaque centrifugation (Sigma Aldrich, Poole, UK). PBMC were resuspended in RPMI (HEPES modification) medium supplemented with 2% fetal calf serum (FCS), 100 IU penicillin/streptomycin, and 2 mM L-glutamine (all from Sigma Aldrich, UK) and stored overnight at 4°C. mDC and pDC were isolated from PBMC by BDCA1 and BDCA4 positive magnetic bead isolation according to the manufacturer's guidelines (Miltenyi Biotec, Germany). mdDC were generated by culturing positively isolated CD14^+^ monocytes (Miltenyi Biotec, Germany) for 7 days in the presence of GM-CSF (150 IU/ml, Immunex, USA) and IL-4 (1000 U/ml, R&D systems, UK). Single donor buffy coats (obtained from the National Blood Bank, Colindale, London) were used as a source of allogeneic healthy NK cells. In brief, PBMC were isolated as outlined above and NK cells were purified by means of CD56 immunomagnetic bead isolation (Miltenyi biotec, Germany) followed by depletion of NKT cells using anti-CD3 Dynal beads (Invitrogen, UK). Cells were then stored at −140°C in FCS containing 10% dimethyl sulfoxide (DMSO, both from Sigma Aldrich, UK) for later use. Purity of the isolated cell subsets (above 90%) was verified by flow cytometry prior to co-culture experiments.

### DC- NK co-cultures

Immature and mature DC subsets were co-cultured with allogeneic NK cells for 24 hours at a ratio of 1∶5. Freshly isolated pDC were used as immature pDC whereas mature pDC were generated by stimulation with CpG ODN 2216 (10 µg/ml, Invivogen, UK). mDC and mdDC were matured with 1 µg/ml LPS. In all experiments, maturation of DC was performed while co-cultured with NK cells thus limiting the ex vivo culture to a total of 24 hours. This was performed in order to minimise cellular death associated with extended in vitro culture periods, especially in cells from viremic HIV-1 patients. Co-culture supernatants were collected and stored at −80°C for cytokine detection by ELISA. Cells were then harvested and labelled for phenotypic analysis or co-cultured with K562 cells for killing/degranulation assays.

### Flow cytometry

Flow cytometric analysis was performed using the following antibodies: anti CD3, CD25, CD69, CD56, CD107a, Lineage, CD11c, CD123, and HLA-DR antibodies (all from Pharmingen or BD biosciences, UK). Cells from co-culture experiments were washed and resuspended in PBS containing 2% FCS, 2 mM EDTA, and 0.05% NaN_3_ (FACS buffer, all reagents from Sigma Aldrich, UK). Cells were stained on ice with the appropriate antibodies for 30 min, washed with FACS buffer, then fixed with 4% paraformaldehyde in PBS (both from Sigma Aldrich, UK). To verify cell purity and to initially identify pDC and mDC, cells were labelled with anti-Lineage, CD123, CD11c, and HLA-DR antibodies. Purity of isolated NK cells was confirmed using CD16, CD56, and CD3 antibodies (all from Pharmingen or BD biosciences, UK). All isolated cells used in this study had a purity of over 90%. Appropriate isotype controls were used to assess non specific binding of the antibodies used. Cells were acquired using a fluorescent-activated cell sorter (FACS-calibur) or an LSR-II (both from BD). Data analysis was performed using Cell Quest Pro software package or FACS-DIVA 5.0.1 (both from BD).

### Degranulation assay

The ability of NK cells to degranulate in response to co-culture with K562 cells was assessed as previously described [32]. In brief, NK cells that had been cultured alone or with DC subsets were further incubated for 4 hours with K562 cells at a 5∶1 ratio in the presence of Brefeldin-A (BFA, 10 µg/ml, Sigma Aldrich), 6 µg/ml monensin (Sigma Aldrich), and anti-human CD107a antibody or its matching isotype control (Pharmingen, BD). Cells were harvested after 4 hours of co-culture and washed in FACS buffer. Cells were then labelled with anti CD3 and CD56 antibodies for 30 mins on ice washed and then fixed with 4% PFA in PBS. Specific staining for CD107a on CD56^+^ CD3^−^ cells (NK cells) was assessed by flow cytometry as described above.

### Cytokine detection

IFN-α and IFN-γ levels in immature and mature DC-NK co-culture supernatants were measured by commercially available ELISA kits. Supernatants were centrifuged and IFN-α and IFN-γ levels measured with human interferon-α multi-subtype ELISA (PBL Interferon Source, USA) and IFN-γ ELISA (R&D Systems, UK) kits. All ELISAs were performed in accordance with the manufacturers' guidelines. ELISA Plates were read on an Anthos ELISA plate reader (ASYS Hitech) at 450 nm. Data was analysed using GraphPad prism 5 (GraphPad Software, San Diego, CA).

### Statistical analysis

Data are expressed as median and IQR or mean ± standard deviation after background subtraction. We used non parametric tests throughout as normality of data distribution could not be tested. We employed the Mann-Whitney U test to determine significance between two un-paired groups and the wilcoxon Rank test to compare paired samples. The Spearman R test was used to determine correlations between two variables. All statistical tests were two-sided and were performed using GraphPad Prism 5 (GraphPad Software, San Diego, CA). p<0.05 was considered statistically significant.

## Results

### Blood DC are depleted in HIV-1 patients

**Figure 1:**
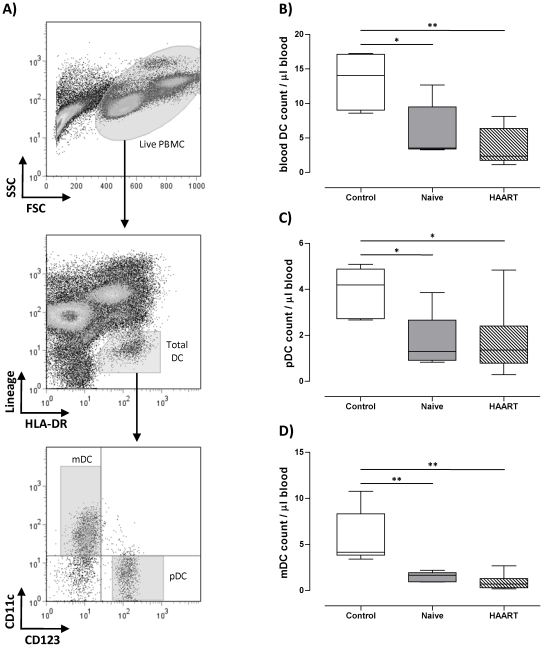
pDC and mDC counts are reduced in HIV-1 infection. A) The gating strategy used to identify blood pDC and mDC within PBMC. B) C) and D) show total DC, pDC, and mDC counts per ul of blood respectively. DC counts were calculated based on their percentages within PBMC, total PBMC live counts (Trypan blue exclusion method), and the volume of blood collected. * and ** indicate P values less than 0.05 and 0.01 respectively (Mann Whitney U test).

We first investigated the effect of HIV-1 infection on circulating blood DC counts. This was achieved by first indentifying the percentages of pDC and mDC within PBMC by flow cytometry on the basis of lack of expression of lineage markers (CD3, CD14, CD16, CD19, CD20, and CD56), expression of HLA-DR, and expression of either CD11c for mDC or CD123 for pDC (Figure 1A). DC counts were then calculated on the basis of their percentages, the total live count of PBMC, and volume of blood collected. As shown in Figure 1B, we confirmed previous observations [26]–[28] that total blood DC numbers are depleted in HIV-1 infected individuals with a median of 3.5 cells/µl (range 3.2 to 12.6 cells/µl, p = 0.03) in therapy-naïve patients, and 2.3 cells/µl (range 1.1 to 8.1 cells/µl, p = 0.002) in HAART-treated patients as compared to a median of 14.1 cells/µl (range 8.6 to 17.3 cells/µl) in healthy controls. Both pDC and mDC counts were reduced in untreated HIV patients with a median of 1.2 cells/µl (range 0.8 to 3.8 cells/µl, p = 0.01), and 1.6 cells/µl (range 0.9 to 2.1 cells/µl, p = 0.004) in pDC and mDC respectively compared to pDC (median of 4.1, range 2.6 to 5 cells/µl) and mDC numbers (median of 4.1 cells/µl, range 3.4 to 10.7 cells/µl) in healthy controls (Figure 1C and D). There was no recovery of pDC and mDC numbers in patients receiving HAART therapy with a median of 1.3 cells/µl (range 0.2 to 4.8 cells/µl, p = 0.01) and 0.7 cells/µl (range 0.1 to 2.6 cells/µl, p = 0.002) respectively (Figure 1C and D).

### pDC from viremic and aviremic HIV-1 patients have impaired NK cell activation capacity

We next sought to investigate the functional competency of pDC to activate NK cells. Since several studies have indicated defects in the NK compartment during HIV-1 infection [22]–[25], we utilised an allogeneic system whereby NK cells were isolated from healthy donors (buffy coats) and co-cultured with pDC that were purified from the different patient groups. This was feasible due to the MHC-non-restricted properties of NK cell activation.

**Figure 2:**
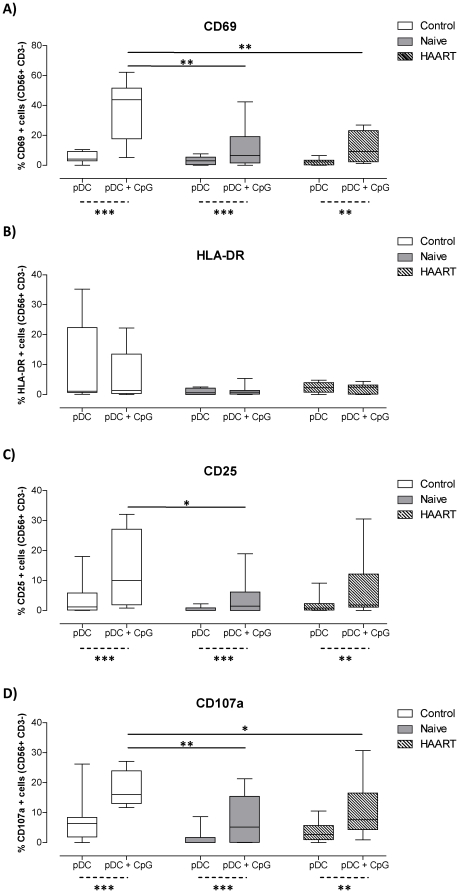
CpG-matured pDC are impaired at activating NK cells. pDC isolated from healthy controls or HIV-1 patients were co-cultured with heterologous NK cells for 24 hours in the presence or absence of CpG DNA (N = 12 for healthy controls (open box plots) and HAART treated patients (striped box plots) and N = 13 for therapy naïve patients(shaded box plots). A), B), and C) show the percentages of CD56^+^ CD3^−^ NK cells expressing CD69, HLA-DR, and CD25 respectively. D) NK cells that were co-cultured with pDC were further co-cultured with K562 cells for 4 hours. Box plots represent the percentages of CD56^+^ CD3^−^ NK cells expressing CD107a. Data was corrected for background expression of the different makers by NK cells that were cultured alone or with K562 cells for the same period of time. *, **, and *** indicate P values less than 0.05 and 0.01 and 0.001 respectively using the Mann Whitney U test (straight lines) or the Wilcoxon paired test (dotted lines).

To monitor NK activation we measured the expression levels of a number of surface receptors by flow cytometry. As shown in Figure 2, co-culture of NK cells with immature pDC that were isolated from healthy controls or HIV-1 patients resulted in minimal activation of NK cells as assessed by lack of expression of CD69, HLA-DR, and CD25. Additionally, there was no or minimal degranulation by NK cells following co-culture with K562 cells, an NK sensitive tumorogenic cell line, or on culture with immature pDC. However, addition of CpG DNA (a TLR-9 ligand) to pDC-NK cell co-cultures from healthy donors resulted in substantial upregulation of CD69 and CD25 by NK cells (Figure 2A and 2C). This was statistically significant with p values equal to 0.001 for both receptors (Wilcoxon paired test) in comparison to immature pDC-NK co-cultures. Similarly, CD107a expression by NK cells following a 4 hour co-culture with K562 cells was increased above background levels by at least 2–3 fold when NK cells were co-cultured with CpG DNA-matured pDC that were purified from healthy donors (p = 0.001, Wilcoxon test, Figure 2D). There was marked impairment in the ability of CpG DNA treated pDC from HIV-1 infected individuals, regardless of their therapy status, to activate NK cells with respect to expression of CD69, CD25, and CD107a compared with healthy controls (Figure 2).

HLA-DR expression by allogeneic NK cells was not up-regulated by co-culture with mature pDC from healthy donors. Interestingly, HLA-DR expression on NK cells was lower when they were co-cultured with stimulated or unstimulated pDC from HIV-1 infected individuals regardless of their therapy status (Figure 2B). Of note, we evaluated the effects of TLR ligands used in this study (CpG DNA and LPS) on NK cell in the absence of DC for control purposes. Both TLR ligands had minimal to no effect with regards to CD69, HLA-DR, CD25, and CD107a expression by NK cells (Figure S1).

### mdDC but not mDC have impaired NK stimulatory functions in HIV-1 patients

**Figure 3:**
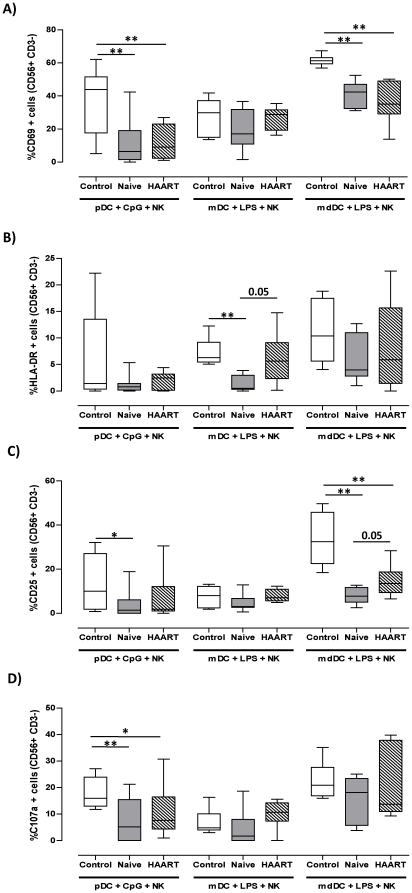
LPS-matured mdDC but not mDC are impaired at activating NK cells. mDC and mdDC from healthy controls (open box plots, n = 6), therapy naïve (filled box plots, n = 7), and HAART treated HIV-1 patients (striped box plots, n = 6) were co-cultured with allogeneic NK cells from healthy donors for 24 hours. A), B) and C) show the percentages of CD56^+^ CD3^−^ NK cells expressing CD69, HLA-DR, and CD25 respectively. D) NK cells were further co-cultured with K562 cells for 4 hours in the presence of CD107a antibody. Box plots represent the percentages of CD56^+^ CD3^−^ NK cells expressing CD107a. The levels of NK activation using CpG DNA matured pDC from the different cohorts are also given for comparative purposes. All data was corrected for background expression of the different makers by NK cells that were cultured alone or with K562 cells for the same period of time. * and ** indicate P values less than 0.05 and 0.01 respectively (Mann Whitney U test).

Since we observed a reduced ability of pDC from HIV-1 infected subjects to stimulate NK cells, we evaluated the functional competency of other DC subsets. We repeated the co-culture experiments using blood mDC and *In vitro* generated DC derived from monocytes (mdDC) using blood from 6 healthy controls and untreated, N01–N07, or HAART treated H01–H06 HIV-1 infected patients (Table 1). Similar to immature pDC, unstimulated mDC and mdDC isolated or generated from healthy controls or HIV-infected individuals lacked the ability to activate allogeneic NK cells (data not shown). When mDC and mdDC from healthy controls were stimulated with LPS, a TLR-4 ligand, then co-cultured with allogeneic NK cells, we observed an increase in CD69 and CD25 expression by NK cells (Figure 3A and C). Up-regulation of these two receptors was more pronounced when mature mdDC were used in comparison to mDC (Figure 3 A and C). Unlike pDC, both LPS-matured mDC and mdDC from healthy individuals were capable of stimulating moderate levels of HLA-DR expression by NK cells (Figure 3 B).

LPS-matured mDC that were isolated from HIV-1 infected patients retained the ability to activate NK cells with regards to CD69, CD25, and CD107a expression (Figure 3 A and C). However, the ability of mDC from therapy naïve patients to stimulate HLA-DR up-regulation by NK cells was severely reduced (mean of 1.3±1.5%, p = 0.001) in comparison to healthy donors (Figure 3B). This function was moderately restored in HAART-treated HIV-1^+^ individuals (mean of 6.1±5.0%), although it did not reach statistical significance in comparison to viraemic patients (p = 0.05, Figure 3 B).

On the other hand, mdDC that were generated from HIV patients showed a diminished ability to activate NK cells with regard to CD69 and CD25 expression. This reduction was statistically significant in comparison to mdDC from healthy individuals (Figure 3A and C). Conversely, there were no statistically significant differences between the different cohorts with regards to the ability of mdDC to induce NK-cell expression of HLA-DR (Figure 3B) and degranulation in response to K562 cells (Figure 3D).

### Reduced secretion of interferon γ by NK cells co-cultured with mature pDC or mature mdDC from HIV-1-infected patients

**Figure 4:**
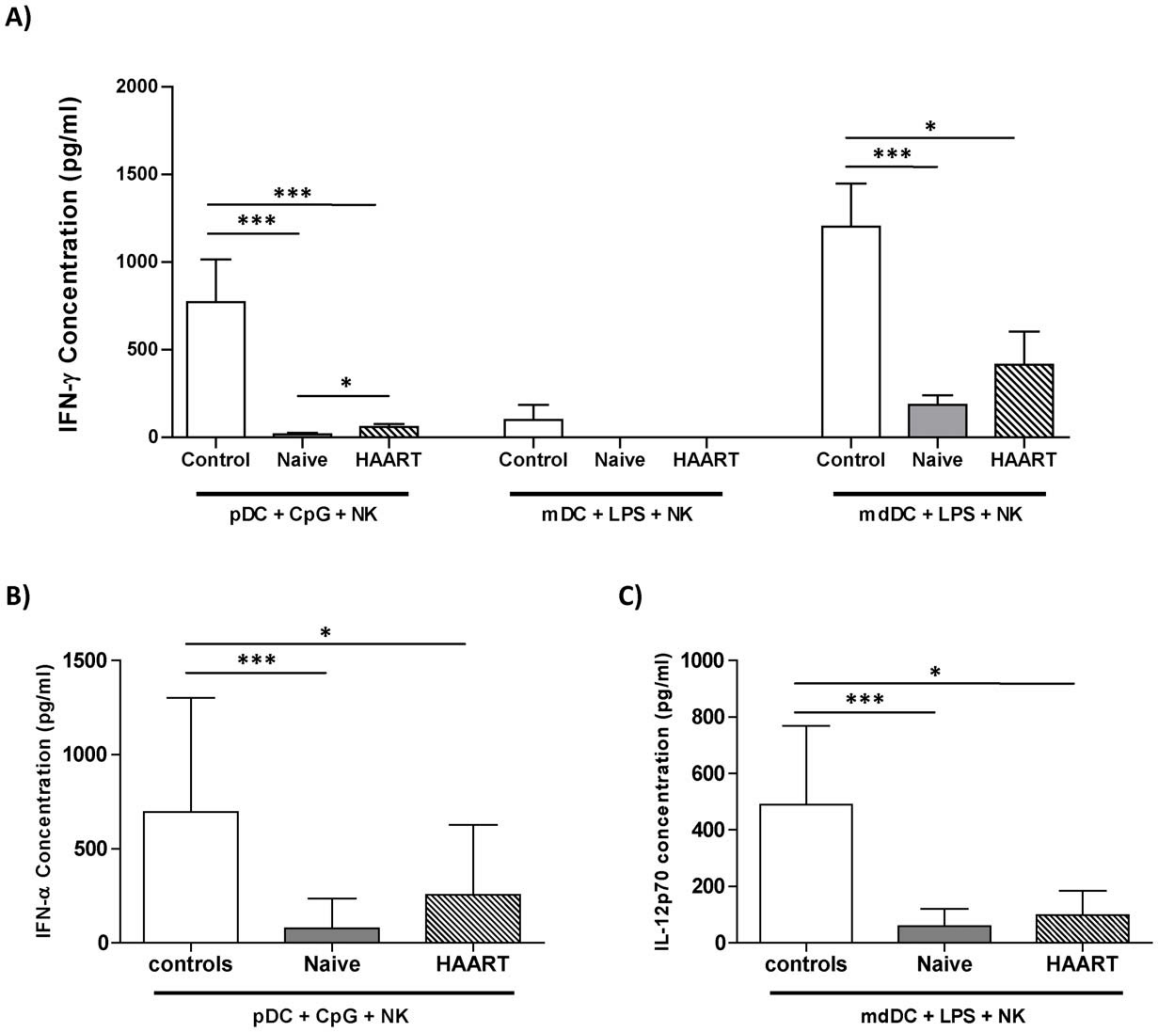
pDC and mdDC-mediated IFN-γ secretion by NK cells is impaired in HIV-1 infection. pDC, mDC, and mdDC from healthy controls (open bars), therapy naïve (filled bars), and HAART treated HIV-1 patients (striped bars) were co-cultures with allogeneic NK cells for 24 hours. The levels of IFN-γ (A), IFN-α (B), and IL-12p70 (C) were measured in cell-culture supernatants by ELISA. * and ** indicate P values less than 0.05 and 0.01 respectively (Mann Whitney U test).

Although the primary function attributed to NK cells is direct killing of tumourogenic and virally-infected cells, a subset of activated NK cells can also secrete large amounts of IFN-γ, a Th1 promoting cytokine with anti-viral properties. We measured the levels of this cytokine in DC-NK cultures from the two patient groups and controls. NK cells that were cultured alone or with immature DC had undetectable IFN-γ levels (below the detection limit of 15 pg/ml, data not shown). In contrast, cultures containing NK cells and CpG-matured pDC or LPS-stimulated mdDC from healthy controls produced significant amounts of IFN-γ (means of 771±243 and 1201±245 pg/ml respectively, Figure 4A). By contrast, mature pDC and mdDC from HIV-1 patients were notably impaired at inducing IFN-γ secretion by NK cells (Figure 4A). There were slightly higher IFN-γ levels in cultures containing mature pDC and mdDC from HAART-treated patients in comparison to untreated individuals (Figure 4A). Nonetheless, these levels remained significantly lower in comparison to healthy controls, indicating that ART therapy does not restore the functional competency of pDC and mdDC to induce IFN-γ production by allogeneic NK cells.

We and others [18], [33], [34] have demonstrated that pDC mediated activation of NK cells is partly mediated through secretion of IFN-α, whereas mdDC stimulate NK cells through an IL-12 dependent mechanism [35]. Therefore, we measured the levels of IFN-α and IL-12p70 in cell culture supernatants containing mature pDC and mdDC respectively. As can be seen in Figure 4B, large amounts of IFN-α were detected in cultures containing CpG DNA-matured pDC from healthy controls. In contrast, pDC from therapy naïve patients secreted significantly lower levels of IFN-α in response to CpG DNA (Figure 4B). pDC from therapy-treated HIV-1 patients produced slightly more IFN-α than that produced by pDC from untreated patients but this was still 2–3 fold lower than pDC from healthy individuals (Figure 4B). Similarly, IL-12p70 secretion by LPS-matured mdDC was found to be severely diminished in HIV-1 patients (Figure 4C) regardless of their therapy status.

Contrary to pDC and mdDC, LPS-matured mDC from the different groups failed to induce NK-mediated IFN-γ secretion. This may reflect their limited ability to secrete IL-12 upon single TLR stimulation as previously described by Napolitani et al [36].

## Discussion

It is well established that HIV infection reduces both DC and NK cell numbers and function [22]–[29]. The objective of the current study was to investigate the ability of DC subsets from HIV-1 infected patients to stimulate various NK cell functions. pDC from HIV-1 seropositive individuals exhibited a defective capacity to induce NK-associated activation markers tested including CD69, an early activation marker expressed by NK cells committed to cytolytic activity [37], [38]; HLA-DR expressed by a subset of activated NK cells [39]; CD25 - associated with increased NK proliferative capacity [38]; and CD107a - a marker of lysosomal granule exocytosis that correlates with NK-mediated cytolytic activity [32]. Although mDC are generally not as potent as mdDC in activation of NK, their NK-stimulatory competency was unaltered during HIV-1 infection, except for HLA-DR up-regulation, whereas mdDC from HIV-1 infected individuals displayed defects in their ability to stimulate CD25 and CD69 up-regulation by NK cells. Notably, pDC and mdDC defects did not correlate with the patients' CD4 T cell counts or plasma viral loads (data not shown), which may indicate that these impairments are not directly associated with disease progression.

Defects, which were not reversed by HAART treatment, may be attributed to DC and not NK cells as we have utilised an allogeneic *In vitro* system in which DC from HIV-1 infected individuals were co-cultured with NK cells from healthy seronegative individuals. Of note, the ability of NK cells from healthy individuals to respond to allogeneic DC was comparable to autologous DC, at least in terms of CD69 expression (data not shown). However we cannot eliminate the possibility that NK cells were able to kill allogeneic DC through recognition of mismatched HLA-C molecules as suggested by a number of *In vitro* [40] and *in vivo* studies [41]. Nonetheless, we observed minimal NK activation in the absence of stimulatory ligands following co-culture with allogeneic immature DC as assessed by expression of CD69, CD25, HLA-DR, and secretion of IFN-γ (Figure 2 and data not shown). This experimental setup has been recently used in a similar study to ours [30].

The effect of HIV-1 infection on circulating DC numbers has been addressed in a number of studies. Both mDC and pDC numbers are significantly lower in chronically infected patients [26], [27]. However, the effect of antiretroviral therapy on restoration of DC numbers in the periphery of HIV-1 infected individuals remains inconclusive. Chehimi et al showed that HAART treatment had no beneficial effect on pDC and mDC percentages and numbers [42], whereas Baron et al and Kamga et al found that HAART treatment during the acute phase of HIV-1 infection restored blood DC counts [28], [43], and Finke et al demonstrated partial reconstitution of DC numbers in chronically infected patients receiving therapy [44]. In the present study ART therapy was found to be ineffective at restoring DC numbers. Notably, DC reduction in the periphery of HIV-1 patients may reflect their migration to secondary lymphoid tissue as shown in primary infection [45].

In our HIV-1 cohorts we observed functional defects in pDC in their ability to stimulate NK cells. In a recent report by Conry et al [30], the authors demonstrated that pDC mediated activation of NK cell secretion of IFN-γ and granzyme B production was impaired during HIV-1 infection. Similarly, Reitano and colleagues showed that CpG-DNA stimulation of PBMC from viraemic and aviraemic HIV-1 patients resulted in lower levels of CD69 expression by NK cells compared to healthy controls [31]. In both studies, impaired pDC-NK interactions in HIV-1 infected individuals were attributed to defects in the pDC and NK cell compartments, with pDC secreting lower amounts of IFN-α and TNF-α upon CpG DNA stimulation [31] whilst NK cells were refractory to type I interferon [30] due to expression of high levels of interferon-inducible genes (IFIGs) [31].

Although pDC from healthy individuals secrete abundant amounts of IFN-α in response to stimulation with TLR-7 and 9 ligands [10], [13], [46], [47], there is a consensus in the literature regarding a diminished type I IFN secretory ability of pDC from HIV-1 infected patients during primary [43], [48] and chronic infection [49]. However, the effect of anti-retroviral treatment on pDC functions remains controversial. On the one hand, it has been suggested that initiation of HAART during acute [43], [48], [50] and chronic [51] HIV-1 infection restores pDC numbers and their ability to secrete IFN-α production upon maturation. On the other hand, Reitano and colleagues showed no reconstitution in pDC-mediated production of IFN-α in HIV-1 patients receiving anti-retroviral therapy [31]. Findings of the current study suggest that HAART treatment did not restore circulating pDC numbers nor did it reverse the refractory nature of pDC to CpG DNA stimulation. However, HAART treated patients who were recruited in this study had received therapy for a median of 5.7 years and it remains plausible that recovery of pDC numbers and function in terms of type I interferon production in these individuals may require a longer period of viral suppressive therapy [52]. Additionally, treated patients recruited in this study had low nadir CD4 counts (median of 163.5, range 0–784 cells/µl) prior to HAART initiation, which may have affected full reconstitution of pDC functions post treatment.

Using unfractionated PBMC, Yonkers et al showed that accessory cell dependent NK cell activation was impaired in HIV-1 patients. This impairment was due to defective mDC-NK interaction and likely a result of the numerical deficiency of mDC associated with HIV-1 infection rather than a defective ability of mDC to stimulate NK cells on a per cell basis [53]. This is in agreement with the finding of the current study where mDC function appears to be intact in HIV-1 infected individuals. However, in the study by Yonkers et al, the authors found that NK-mediated secretion of IFN-γ was dependent on mature mDC and partly dependent on IL-12. Additionally, an earlier report by Geroza et al indicated that mDC from healthy individuals were able to activate NK cells through an IL-12-dependent mechanism [18]. Although these observations seem to contradict the findings of this report where we observed no IL-12p70 production by LPS-matured mDC, a possible explanation for this discrepancy is differences in the stimuli used to mature mDC. Both Yonkers et al [53] and Geroza et al [18] utilised synthetic Poly-IC which signals through TLR-3 whereas we used LPS which activates mDC via TLR-4. In our study, mature mDC failed to induce IFN-γ secretion by NK cells possibly due to their inability to secrete IL-12p70 in response to LPS, whereas Yonkers et al detected IL-12 in their cell culture supernatants, albeit the levels were extremely low (less than 25 pg/ml). Our observation that mDC did not secrete IL-12 in response to TLR-4 stimulation is consistent with a previous report [36] indicating that a combination of TLR agonists are required for synergistic activation of IL-12 p70 production by human blood mDC.

Little is known about the effect of HIV-1 infection on monocyte-derived DC (mdDC) with respect to their ability to activate NK cells. Early studies conducted in seronegative controls suggest that unlike pDC-mediated activation of NK cells which is dependent on Type I interferon and cellular contact [18], [33], mdDC activate NK cell functions primarily through secretion of IL-12p70 [17], [19]. Thus, it is reasonable to assume that the diminished ability of mdDC from HIV-1 patients to stimulate NK cells may be completely or at least partially due to their impaired IL-12 secretory potential. Indeed, we observed a significant positive correlation between the levels of IL-12p70 in mdDC-NK co-culture supernatants and IFN-γ secretion by NK cells in all cohorts (data not shown). Other studies have addressed the capacity of mdDC from HIV-1 patients to secrete the bioactive form of IL-12. For instance, Sacchi and co-workers demonstrated that monocytes from both therapy-naive and treated HIV-1 patients differentiate into CD1a^−^ dendritic cells with a defective capacity to secrete IL-12p70 in response to *In vitro* stimulation with LPS [54]. We have recently assessed the functional competency of mdDC from HIV-1 infected individuals to stimulate T cell proliferation and demonstrated that although mdDC express normal levels of co-stimulatory molecules upon LPS maturation, they produced significantly lower amounts of IL-12p70 [55]. Similarly, Mavillio et al showed a significant reduction in the ability of mdDC from viraemic individuals to secrete IL-12p70 with a trend towards recovery on therapy [56]. This was coupled with a reduction in the ability of mdDC from viremic patients to induce IFN-γ secretion by NK cells [56]. The aforementioned reports are consistent with the findings presented in Figure 4. Interestingly, in the study by Mavillio et al [56], mdDC from viremic patients were also impaired at inducing proliferation of both autologous and allogeneic NK cells. This is consistent with our findings regarding a diminished expression of CD25, a marker associated with proliferation (Figure 3). However, we observed a partial increase in CD25 expression by NK cells that were co-cultured with mdDC from aviremic treated patients, though this remained signficantly less than that observed with mdDC from healthy donors. The effect of HAART on mdDC function in this respect, as well as IFN-γ secretion, in our cohort is inconsistent with the findings of Mavillio et at where they observed full recovery of function. This may reflect differences in the type of treatment, length of therapy, or the patients' nadir CD4 counts.

As previously mentioned, DC-NK interactions are not unidirectional as it is known that activated NK cells can either lyse immature DC or induce their maturation depending on NK-DC ratios [20]. It is thought that through these two mechanisms, NK cells can either provide a selection mechanism for DC that are competent at priming T cells or provide a negative regulatory mechanism of on-going inflammatory responses, whilst NK-mediated induction of DC maturation in the absence of danger signals may be important in the initiation of adaptive immune responses, particularly in anti-tumour immunity. Although the current report has not directly addressed the effects of NK cells on DC maturation, it is likely that the decreased levels of IFN-γ secretion by NK cells seen in HIV-1 patients (Figure 4) may result in lower levels of DC maturation as suggested by Vitale et al [57]. However, further experiments would be required to fully address these points. With regards to NK-mediated lysis of immature DC, two early studies [56], [58] have demonstrated an impaired ability of NK cells from HIV-1 infected individuals to kill autologous immature mdDC. However, HAART therapy was shown to restore this function in one study [56] whilst Tasca et al found that this function was conserved in early infection but lost in chronically infected individuals regardless of their therapy status [58]. There is no data thus far as to whether NK-mediated killing of naturally occurring DC (pDC and mDC) is affected during HIV-1 infection and how this may impact on HIV-1 spreading and pathogenesis, further experiments are needed to elucidate this point. In addition, future studies should dissect the precise mechanisms by which HIV-1 affects this innate NK-DC network.

In conclusion, findings presented in this study suggest that HIV-1 infection selectively impairs pDC and mdDC-mediated NK activation. Since NK cells play a pivotal role in anti-HIV-1 immune responses, our results may have implication for future design of vaccines and immune-based therapies. Additionally, the observation that HAART had little effect on reconstitution of these innate functions further implies that alternative therapeutic strategies are required for an enhanced immune recovery.

## Supporting Information

Figure S1 **Effect of LPS and CpG DNA on NK cell activation.** Purified NK cells were cultured for 24 hours either alone (negative control), or in the presence of CpG DNA, LPS, or phorbol 12-myristate 13-acetate (PMA) and ionomycin (positive control). Cells were harvested and stained for CD69 (A), HLA-DR (B), and CD25 (C). NK cells were also co-incubated with K562 cells for 4 hours and CD107a is shown in (D). Filled Histograms and dotplots represent expression levels by CD56+ CD3- cells from a representative sample. Dotted lines and quadrants indicate nonspecific staining using the appropriate isotype controls. Scatter plots (right) represent cumulative data from all samples and batches of NK cells used in this study.
(TIF)
