# Supplementary figures and images for: Loss of NK Stimulatory Capacity by Plasmacytoid and Monocyte-Derived DC but Not Myeloid DC in HIV-1 Infected Patients
Source: PLoS One. 2011 Mar 8;6(3):e17525. doi: 10.1371/journal.pone.0017525 (PMC3050890; doi:10.1371/journal.pone.0017525)

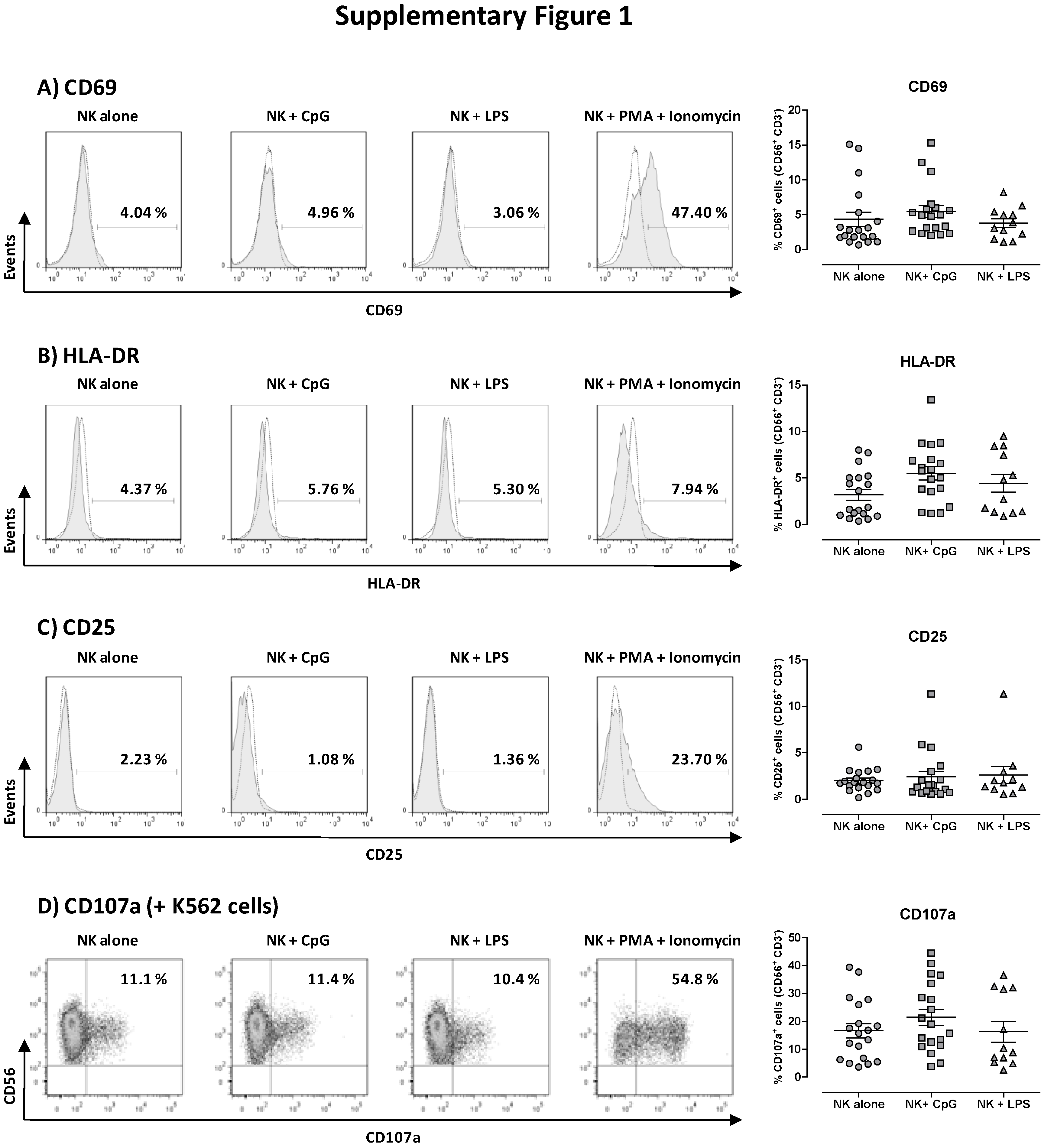

Supplement: Figure S1 — Effect of LPS and CpG DNA on NK cell activation. Purified NK cells were cultured for 24 hours either alone (negative control), or in the presence of CpG DNA, LPS, or phorbol 12-myristate 13-acetate (PMA) and ionomycin (positive control). Cells were harvested and stained for CD69 (A), HLA-DR (B), and CD25 (C). NK cells were also co-incubated with K562 cells for 4 hours and CD107a is shown in (D). Filled Histograms and dotplots represent expression levels by CD56+ CD3- cells from a representative sample. Dotted lines and quadrants indicate nonspecific staining using the appropriate isotype controls. Scatter plots (right) represent cumulative data from all samples and batches of NK cells used in this study. (TIF) [file pone.0017525.s001.tif]
